# Association of Cortisol Levels With Neuropsychiatric Functions: A Mendelian Randomization Analysis

**DOI:** 10.3389/fendo.2019.00564

**Published:** 2019-08-16

**Authors:** Xiang Zhou, Nidan Qiao

**Affiliations:** ^1^Department of Neurosurgery, Huashan Hospital, Fudan University, Shanghai, China; ^2^Neuroendocrine Unit, Massachusetts General Hospital, Harvard Medical School, Boston, MA, United States

**Keywords:** Cushing's syndrome, depression, neuroendocrinology, single nucleotide polymorphisms, cortisol

## Abstract

**Aim:** The conflicting evidence as to whether a real association exists between cortisol levels and depression lends support to adopting a Mendelian randomization approach to investigate whether cortisol levels have a causal effect with depression.

**Methods:** Single nucleotide polymorphisms (SNPs) associated with serum morning plasma cortisol level and salivary cortisol level from CORNET consortium (12,597 participants) were proposed as instrumental variables. The primary outcome was depression, and the secondary outcomes were neuroticism and cognitive performance. Summary-level statistics were extracted from the Social Science Genetic Association Consortium including the United Kingdom Biobank cohort (105,739 subjects). Multiple analysis methods (inverse-variance weighted method, max likelihood method, weighted median estimator, model-based estimation, heterogeneity-penalized method, and MR-Egger regression) were applied to test the stability of the summary causal estimate.

**Results:** Weighted median analysis estimated that the effect of serum morning cortisol on depression score was 0.027 per standard deviation increase of cortisol (95% CI, 0.000–0.054; *p* = 0.043). Other sensitivity analysis suggested similar results suggesting the result was robust. No evidence of pleiotropy (MR-Egger intercept, −0.002; *p* = 0.739) was observed. The effect of serum cortisol on neuroticism was 0.030 (95% CI, 0.008–0.052; *p* = 0.006) by weighted median estimator. None of the methods observed the effect of serum cortisol level on cognitive function. As for the effect of salivary cortisol level, no method obtained a *p*-value lower than 0.05 in any of the outcomes.

**Conclusion:** Mendelian randomization analysis provided evidence that a genetic predisposition to higher serum morning cortisol level was associated with increased depression score.

## Introduction

One of the most common psychiatric diseases in Cushing's syndrome is major depression with the prevalence of 50–81% (1). Some studies have reported an improvement in neuropsychiatric disorders after the resolution of hypercortisolism, suggesting the causal effect of cortisol on depression (2).

Morning serum cortisol and salivary cortisol levels are two diagnostic tools for Cushing's syndrome. Studies about the causaleffect of cortisol levels on depression varied among studies. The positive association between cortisol measures with overall depressive symptoms was observed in the TRAILS cohort (3).

Midnight salivary cortisol was associated with self-reported depression in patients with type 1 diabetes (4). Caparros-Gonzalez et al. (5) used hair cortisol level as a positive predictor for postpartum depression. On the contrary, several studies observed a negative association of cortisol levels and depression. Lower cortisol awakening response correlated with subclinical depression in young subjects (6). Rhebergen et al. (7) found a blunted cortisol awakening response in older people with the depressive disorder. Low salivary cortisol concentration is a risk factor of depression recurrence in another 2-year follow-up study (8).

Neuroticism and cognitive performance are two other neuropsychiatric assessment in patients with Cushing's syndrome. Increased prevalence of neuroticism was observed in patients with Cushing's disease but without knowing the effect size (9, 10). Studies on cognitive performance in chronic exposure of cortisol were controversial with both positive and negative results (11, 12).

**Figure 1:**
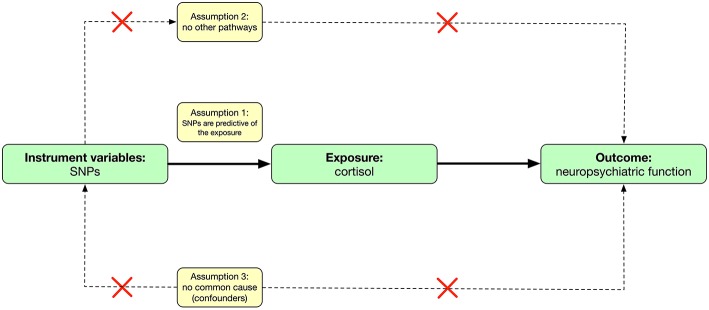
Causal diagram and three assumptions in mendelian randomization.

Mendelian randomization is an analytic method that involves finding genetic proxies for a target exposure and then testing the association with the outcome (13) (Figure 1). This approach avoids some of the limitations of observational studies (free from measured or unmeasured confounding) and is not affected by the disease, thereby avoiding reverse causation bias (14). On the other hand, summary-level data of large genome-wide association studies (GWAS) are increasingly available and allow the use of combinations of single nucleotide polymorphisms (SNPs) in Mendelian randomization analyses.

The conflicting evidence of whether a real association exists between cortisol levels and depression and if so, the magnitude of that association lends support to adopting a Mendelian randomization approach. We hypothesized that cortisol levels have a causal effect on depression as well as neuroticism and cognitive performance. We conducted a Mendelian randomization study, using summary-level data from publicly available GWAS to investigate the causal relationship of cortisol levels with depression as well as neuroticism and cognitive performance.

## Methods

### Study Design and Data Sources

The mendelian randomization approach used in this study builds on three assumptions (13, 15) (Figure 1). First, the genetic variants [instrumental variables: single nucleotide polymorphisms (SNPs)] are predictive of the exposure (cortisol levels); second, the genetic variants are only associated with the outcome (neuropsychiatric functions) through the variants-exposure-outcome pathway; third, the variants do not share common causes with the outcomes. This study involved analysis of publicly available, summary-level data; specific ethical review and informed consent were obtained in all of the original studies. Ethical approval of this study was not required due to not involving human subjects.

### Instrumental Variables

CORNET consortium was a genome-wide association study (GWAS) for genetic association with serum morning cortisol and salivary cortisol levels (16, 17). The GWAS was conducted in 12,597 individuals of European ancestry (established in a cohort of 74.9 ± 5.7 years old with 45.4% of male and validated in a cohort of 60.9 ± 5.7 years old with 77.7% of male). Eighteen SNPs associated with serum morning cortisol level at the genome-wide significance level (*p* < 5.0 × 10^−8^) in the discovery cohorts (Supplement Table 1). Effect alleles in these SNPs associated with at least 0.07 standard deviation (SD) increase of serum morning cortisol level in the log scale.

Though no SNPs associated with salivary cortisol levels at the genome-wide significance level were identified, we still extracted two SNPs at the significance level of *p* < 5.0 × 10^−7^ (Supplement Table 2). Effect alleles in these two SNPs were associated with at least 0.12 SD increase of salivary cortisol level in the log scale.

### Outcomes

The primary outcome was depression. We extracted summary-level statistics for the associations of the cortisol-associated SNPs from the study conducted by Okbay et al. (18) in 105,739 subjects. The population came from UK Biobank (UKB) cohorts. The outcome used a continuous measure by combining responses to two questions. The first question was: “Over the past 2 weeks, how often have you felt down, depressed or hopeless?” The second question was: “Over the past 2 weeks, how often have you had little interest or pleasure in doing things?” Answers were categorized into: (1) “Not at all,” (2) “Several days,” (3) “More than half the days,” and (4) “Nearly every day.”

The secondary outcomes were neuroticism and cognitive performance. We extracted summary-level statistics of neuroticism from a population of 168,105 subjects with the population coming from the Genetics of Personality Consortium (GPC) and UKB data (18). The outcome used in UKB cohort was summing response of a 12-item version of the Eysenck Personality Inventory (mean: 4.16, SD: 3.23). The outcome used in GPC was harmonized different neuroticism batteries. The summary-level statistics of cognitive performance were obtained from a recently published study (19) using the respondent's score on a test of verbal cognition.

### Sensitivity Analyses

We used multiple analysis methods (inverse-variance weighted method, max likelihood method, weighted median estimator, model-based estimation, and heterogeneity-penalized method) to test the stability of the summary causal estimate. We used MR-Egger regression to test pleiotropy. In the absence of statistical evidence for horizontal pleiotropy (the intercept from MR-Egger not differed from zero), we used the conventional Mendelian randomization analyses as they retain greater power (20). We further examined the stability of the summary causal estimate by excluding SNPs with high linkage disequilibrium with each other from the instrumental variables.

### Statistical Analysis

The SD for cortisol in the GWAS cohorts was around 200 nmol/L, with a SD of 1 for depression symptom score. Assuming that the lowest observational causal effect is 0.003 depression score increase per 1 nmol/L cortisol increase based on observational studies (21, 22), and also assuming that the genetic instrument explains only 1% of variation in cortisol level (16, 17), the calculated power of this Mendelian randomization study in 100,000 subjects was 35% (23). If we assume that the causal effect is 0.01 depression score increase per 1 nmol/L cortisol increase, the calculated power will be 100%.

SNPs were matched across the data sources by assigning them to the same effect allele. The association of each SNP with primary or secondary outcomes was weighted by its association with cortisol levels. The effect of cortisol levels on primary or secondary outcomes was scaled per SD increase. Statistical tests for the Mendelian randomization analyses were considered significant at *p* = 0.05. All analyses were conducted in R version 3.2.5.

## Results

In the UKB cohort (British population-based cohort focused on individuals in the age range 40–69) for depression, the average score of the first question was 1.29 ± 0.61, the average score of the second question was 1.27 ± 0.60. The combination of those two questions achieved an average score of 2.56 ± 1.09 (18).

**Figure 2:**
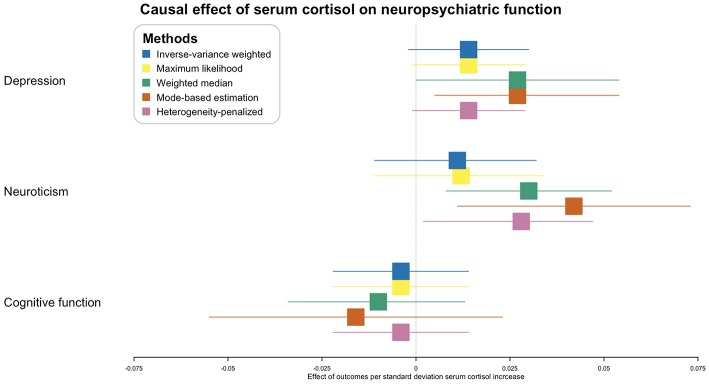
Causal effect of serum morning cortisol level on neuropsychiatric functions. X-axis represents the effect of per standard deviation of serum cortisol increase on the neuropsychiatric outcomes. Boxes represent point estimations of the effect with the 95% confidence interval. Different colors represent different statistical methods.

**Figure 3:**
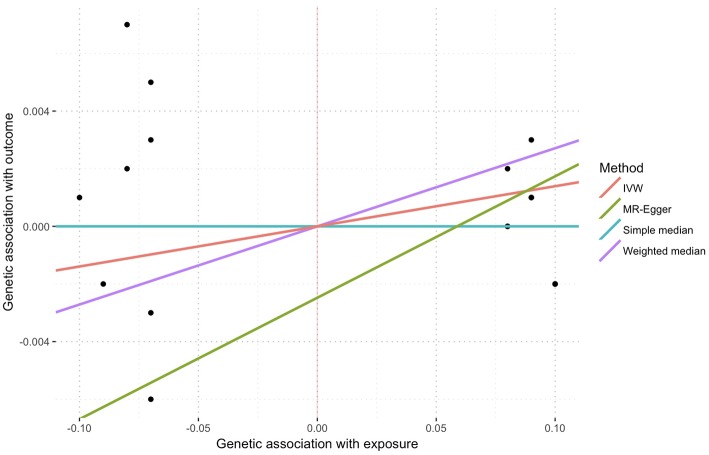
Sensitivity plot of genetic association with exposure vs. with outcome using multiple methods. Points represent SNPs. There was no evidence of pleiotropy represent by the intercept of MR-Egger (−0.002, *p* = 0.739).

In the inverse-variance weighted analysis, the effect of serum morning cortisol on depression score was 0.014 per SD increase of cortisol (95% CI, −0.002–0.030; *p* = 0.081) (Figure 2). Max likelihood method obtained almost the same estimation and confidence interval. Weighted median estimated the effect to be 0.027 (95% CI, 0.000–0.054; *p* = 0.043). Model-based estimation also obtained almost the same estimation and confidence interval with weighted median estimator. The effect was 0.014 (95% CI, −0.001–0.029) using the heterogeneity-penalized method. The association between serum cortisol levels and depression score was consistent in MR-Egger regression, although with a wider confidence interval (0.042, [95% CI, −0.125–0.209]). There was no evidence of pleiotropy (MR-Egger intercept, −0.002; *p* = 0.739, Figure 3). No heterogeneity was observed between the estimates from different methods (*I*^2^ = 0%, *p* = 0.76 for heterogeneity).

Inverse-variance weighted and max likelihood estimation obtained null results (0.011, [95% CI, −0.011–0.032, *p* = 0.322] and 0.012, [95% CI, −0.011–0.034, *p* = 0.563], respectively) regarding the effect of serum cortisol on neuroticism (Figure 2). The effect using weighted median estimator, model-based estimation and heterogeneity-penalized method were 0.030 (95% CI, 0.008–0.052, *p* = 0.006), 0.042 (95% CI, 0.011–0.073, *p* = 0.008) and 0.028 (95% CI, 0.002–0.047), respectively. None of the method observed effect of serum cortisol level on cognitive function, *p*-values ranged from 0.277 to 0.674 (Figure 2).

**Figure 4:**
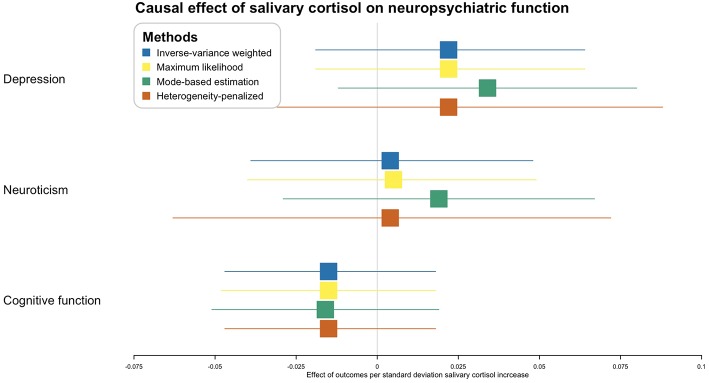
Causal effect of salivary cortisol level on neuropsychiatric functions. X-axis represents the effect of per standard deviation of salivary cortisol increase on the neuropsychiatric outcomes. Boxes represent point estimations of the effect with the 95% confidence interval. Different colors represent different statistical methods.

As for the effect of salivary cortisol level, no method obtained a *p*-value lower than 0.05 in any of the outcomes (Figure 4).

In the sensitivity analysis excluding SNPs with high linkage disequilibrium with each other, which provided 8 SNPs in the remaining instrumental variables, the effect of serum morning cortisol level on depression score using weighted median estimator was 0.026 (95% CI, −0.002–0.055, *p* = 0.072, Supplement Figure 1).

## Discussion

To reveal the causal effects of cortisol levels on the depression, we conducted this Mendelian randomization analysis using public available summary-level data in Caucasian subjects. We observed positive associations of serum morning cortisol with self-report depression score and neuroticism score.

Among three assumptions of Mendelian randomization, using serum cortisol associated SNPs as instrumental variables in this study probably meet the first assumption. Effect alleles in each SNP were associated with at least 0.07 SD increase of cortisol level. Proposed salivary cortisol associated SNPs as instrumental variables may violate this assumption due to failing the genome-wide significant level. This may explain that no causal effect was observed between salivary cortisol and the outcomes.

The second Mendelian randomization assumption may be violated when instrumental variables display associations with the outcome through pathways that are distinct to the one from exposure through to outcome (15). Though the second assumption was not verifiable, we adopted several approaches to minimize the originated bias. We evaluated each cortisol-associated SNP for pleiotropic associations with potential known confounders, including body mass index and diabetes. None of these SNPs had pleiotropic associations at the Bonferroni-corrected significance threshold [(*p* < 0.05)/18 SNPs = 0.003, Supplement Table 3]. We used MR-Egger regression as one of the sensitivity tests, which provided a valid test of pleiotropy (no evidence of pleiotropy) and a valid test of the causal null hypothesis. The slope of MR-Egger regression (0.042, Figure 4) provided a consistent estimate of the true causal effect (20).

The third mendelian randomization assumption may also be violated when instrumental variables and outcomes share common causes (15). This may be the case in population stratification and linkage disequilibrium. We restricted the study in the frame of the European population, which minimized the risk of population stratification. We also searched SNPs in the same linkage disequilibrium block but not included in the instrumental variables (261 SNPs). No association of those SNPs with the outcomes (Supplement Table 4) was observed at the Bonferroni-corrected significance level [(*p* < 0.05)/261 SNPs = 0.0002]. In the sensitivity analysis, we removed SNPs with high association with each other. The point estimations almost unchanged, though the confidence intervals of most methods included the null hypothesis, which was caused by decreased number of instrumental variables.

SNPs associated with serum cortisol level and saliva cortisol level were established and validated in two different cohorts with different ages and different gender proportion, which suggests age and gender do not influence the association between SNPs and cortisol levels. On the other hand, UKB is a relatively younger cohort comparing to the two discovering cohorts, but we argue that even though age is associated with the outcomes, there is no evidence that age would be associated with the SNPs.

We used five different statistical methods to estimate the causal effect because either method had its own advantage. Inverse-variance weighted method is biased when one genetic variant is an invalid instrument variable (24). Weighted median gives consistent estimation when 50% of the genetic variants is valid (24). Maximum likelihood method has been encouraged especially in weak instruments due to the unbiased estimation (25). The model base estimation and heterogeneity-penalized method presented less bias and lower type-I error rates than other methods under the null (26, 27). MR-Egger method is consistent even when 100% of genetic variants are invalid but has wider confidence interval than inverse-variance weighted or median-based methods (24).

In patients with Cushing's syndrome, morning serum cortisol level may increase by 10 standard deviations in certain patients, which may result in the depression score increase by approximately 0.2 (roughly half day of depression according to the question and answer in the phenotype). The finding from this study supports results from several observational studies showing a positive association of serum cortisol levels with risk of depression (3–5). Different scales used in the outcome among studies increase the difficulty of comparison. Findings from observational studies further show that recovery of hypercortisolemia is associated with recovery of depression (1, 28). The positive association between serum cortisol and neuroticism also supports previous observational studies (9, 10, 29–31).

This was the first study to investigate the causal relation of cortisol levels with depression using a very large sample and a method that may allow for unbiased estimates. The results in this study were robust with multiple sensitivity analysis indicating that confounding is unlikely to explain the observed association.

This study has some limitations. First, although we used several approaches in an attempt to rule out pleiotropy, it was still possible that the association of SNPs and depression were through other pathways. A shared genetic basis between SNPs and depression cannot be ruled out either. Second, outcomes derived from self-report answers. Although the continuous outcomes were more likely to increase power than binomial diagnostic data in this study, their clinical use has not been validated. The DSM diagnosis can be used for depression in future studies. Third, only two SNPs were associated with salivary cortisol levels in the original GWAS which made the analysis not robust on the effect of salivary cortisol levels. Besides, neither individual-level SNP data nor a replication data set with a similarly large number of depression cohort were not available. Fourth, this analysis was restricted to individuals of European ancestry, thus hindering the generalizability in the whole population. The results were derived from only statistical analyses and thus, they have to be presented with more cautions. Future studies may investigate cortisol level and genotyping from subjects with depression from the UKB cohorts to see if the genetic variants confirm the association of the cortisol exposure with the disease.

## Conclusions

Mendelian randomization analysis provided evidence that a genetic predisposition to higher serum morning cortisol level was associated with increased depression score.

## Author Contributions

NQ: conception and design of the study. XZ: acquisition and analysis of data and drafting the manuscript.

### Conflict of Interest Statement

The authors declare that the research was conducted in the absence of any commercial or financial relationships that could be construed as a potential conflict of interest.

## References

[1] Pivonello R Isidori AM De Martino MC Newell-Price J Biller BMK Colao A. Complications of Cushing's syndrome: state of the art. Lancet Diab Endocrinol. (2016) 4:611–29. 10.1016/S2213-8587(16)00086-3 27177728

[2] Pivonello R Simeoli C De Martino MC Cozzolino A De Leo M Iacuaniello D Neuropsychiatric disorders ex Cushing's syndrome. Front Neurosci. (2015) 9:129 10.3389/fnins.2015.00129 25941467 PMC4403344

[3] Dietrich A Ormel J Buitelaar JK Verhulst FC Hoekstra PJ Hartman CA. Cortisol in the morning and dimensions of anxiety, depression, and aggression in children from a general population and clinic-referred cohort: an integrated analysis. The TRAILS study. Psychoneuroendocrinology. (2013) 38:1281–98. 10.1016/j.psyneuen.2012.11.013 23237815

[4] Melin EO Thunander M Landin-Olsson M Hillman M Thulesius HO. Depression, smoking, physical inactivity and season independently associated with midnight salivary cortisol in type 1 diabetes. BMC Endocr Disord. (2014) 14:75. 10.1186/1472-6823-14-75 25224993 PMC4236572

[5] Caparros-Gonzalez RA Romero-Gonzalez B Strivens-Vilchez H Gonzalez-Perez R Martinez-Augustin O Peralta-Ramirez MI. Hair cortisol levels, psychological stress and psychopathological symptoms as predictors of postpartum depression. PLoS ONE. (2017) 12:e0182817. 10.1371/journal.pone.0182817 28846691 PMC5573300

[6] Dedovic K Engert V Duchesne A Lue SD Andrews J Efanov SI. Cortisol awakening response and hippocampal volume: vulnerability for major depressive disorder? Biol Psychiatry. (2010) 68:847–53. 10.1016/j.biopsych.2010.07.025 20864090

[7] Rhebergen D Korten NCM Penninx BWJH Stek ML van der Mast RC Oude Voshaar R. Hypothalamic-pituitary-adrenal axis activity in older persons with and without a depressive disorder. Psychoneuroendocrinology. (2015) 51:341–50. 10.1016/j.psyneuen.2014.10.005 25462906

[8] Grynderup MB Kolstad HA Mikkelsen S Andersen JH Bonde JP Buttenschøn HN. A two-year follow-up study of salivary cortisol concentration and the risk of depression. Psychoneuroendocrinology. (2013) 38:2042–50. 10.1016/j.psyneuen.2013.03.013 23597874

[9] Kelly WF Kelly MJ Faragher B. A prospective study of psychiatric and psychological aspects of Cushing's syndrome. Clin Endocrinol. (1996) 45:715–20. 9039337 10.1046/j.1365-2265.1996.8690878.x

[10] Dimopoulou C Ising M Pfister H Schopohl J Stalla GK Sievers C. Increased prevalence of anxiety-associated personality traits in patients with Cushing's disease: a cross-sectional study. Neuroendocrinology. (2013) 97:139–45. 10.1159/000338408 22572774

[11] Forget H Lacroix A Bourdeau I Cohen H. Long-term cognitive effects of glucocorticoid excess in Cushing's syndrome. Psychoneuroendocrinology. (2016) 65:26–33. 10.1016/j.psyneuen.2015.11.020 26708069

[12] Heald A Parr C Gibson C O'driscoll K Fowler H. A cross-sectional study to investigate long-term cognitive function in people with treated pituitary Cushing's disease. Exp Clin Endocrinol Diab. (2006) 114:490–7. 10.1055/s-2006-924332 17115346

[13] Thanassoulis G O'Donnell CJ. Mendelian randomization: nature's randomized trial in the post-genome era. JAMA. (2009) 301:2386–8. 10.1001/jama.2009.812 19509388 PMC3457799

[14] Haycock PC Burgess S Wade KH Bowden J Relton C Davey Smith G. Best (but oft-forgotten) practices: the design, analysis, and interpretation of Mendelian randomization studies. Am J Clin Nutr. (2016) 103:965–78. 10.3945/ajcn.115.118216 26961927 PMC4807699

[15] Smith GD Ebrahim S “Mendelian randomization”: can genetic epidemiology contribute to understanding environmental determinants of disease? Int J Epidemiol. (2003) 32:1–22. 10.1093/ije/dyg070 12689998

[16] Velders FP Kuningas M Kumari M Dekker MJ Uitterlinden AG Kirschbaum C. Genetics of cortisol secretion and depressive symptoms: a candidate gene and genome wide association approach. Psychoneuroendocrinology. (2011) 36:1053–61. 10.1016/j.psyneuen.2011.01.003 21316860 PMC3940151

[17] Neumann A Direk N Crawford AA Mirza S Adams H Bolton J. The low single nucleotide polymorphism heritability of plasma and saliva cortisol levels. Psychoneuroendocrinology. (2017) 85:88–95. 10.1016/j.psyneuen.2017.08.011 28843169

[18] Okbay A Baselmans BML De Neve J-E Turley P Nivard MG Fontana MA Genetic variants associated with subjective well-being, depressive symptoms, and neuroticism identified through genome-wide analyses. Nat Genet. (2016) 48:624–33. 10.1038/ng.3552 27089181 PMC4884152

[19] Lee JJ Wedow R Okbay A Kong E Maghzian O Zacher M. Gene discovery and polygenic prediction from a genome-wide association study of educational attainment in 1.1 million individuals. Nat Genet. (2018) 50:1112–21. 10.1038/s41588-018-0147-3 30038396 PMC6393768

[20] Bowden J Davey Smith G Burgess S. Mendelian randomization with invalid instruments: effect estimation and bias detection through Egger regression. Int J Epidemiol. (2015) 44:512–25. 10.1093/ije/dyv080 26050253 PMC4469799

[21] Xu Y Yao S Wei H Zhu X Yu M Li Y. Application value of selected serum indicators in the differential diagnosis of geriatric depression and transient depressive state. NDT. (2018) 14:459–65. 10.2147/NDT.S152247 29445283 PMC5810520

[22] Doolin K Farrell C Tozzi L Harkin A Frodl T O'Keane V. Diurnal hypothalamic-pituitary-adrenal axis measures and inflammatory marker correlates in major depressive disorder. Int J Mol Sci. (2017) 18:2226. 10.3390/ijms18102226 29064428 PMC5666905

[23] Brion M-JA Shakhbazov K Visscher PM. Calculating statistical power in Mendelian randomization studies. Int J Epidemiol. (2013) 42:1497–501. 10.1093/ije/dyt179 24159078 PMC3807619

[24] Bowden J Davey Smith G Haycock PC Burgess S. Consistent estimation in Mendelian randomization with some invalid instruments using a weighted median estimator. Genet Epidemiol. (2016) 40:304–14. 10.1002/gepi.21965 27061298 PMC4849733

[25] Burgess S Small DS Thompson SG. A review of instrumental variable estimators for Mendelian randomization. Stat Methods Med Res. (2017) 26:2333–55. 10.1177/0962280215597579 26282889 PMC5642006

[26] Hartwig FP Davey Smith G Bowden J. Robust inference in summary data Mendelian randomization via the zero modal pleiotropy assumption. Int J Epidemiol. (2017) 46:1985–98. 10.1093/ije/dyx102 29040600 PMC5837715

[27] Burgess S Zuber V Gkatzionis A Rees JMB Foley C Improving on a modal-based estimation method: model averaging for consistent and efficient estimation in Mendelian randomization when a plurality of candidate instruments are valid. Epidemiology. (2017) 2017:1–28. 10.1101/175372 PMC6124628 29846613

[28] Dorn LD Burgess ES Friedman TC Dubbert B Gold PW Chrousos GP. The longitudinal course of psychopathology in Cushing's syndrome after correction of hypercortisolism. J Clin Endocrinol Metab. (1997) 82:912–9. 10.1210/jcem.82.3.3834 9062506

[29] Rietschel L Streit F Zhu G McAloney K Kirschbaum C Frank J. Hair cortisol and its association with psychological risk factors for psychiatric disorders: a pilot study in adolescent twins. Twin Res Hum Genet. (2016) 19:438–46. 10.1017/thg.2016.50 27374135

[30] Garcia-Banda G Chellew K Fornes J Perez G Servera M Evans P. Neuroticism and cortisol: pinning down an expected effect. Int J Psychophysiol. (2014) 91:132–8. 10.1016/j.ijpsycho.2013.12.005 24361230

[31] Hauner KKY Adam EK Mineka S Doane LD DeSantis AS Zinbarg R. Neuroticism and introversion are associated with salivary cortisol patterns in adolescents. Psychoneuroendocrinology. (2008) 33:1344–56. 10.1016/j.psyneuen.2008.07.011 18809259 PMC2617715

