# Supplementary material for: Association of Cortisol Levels With Neuropsychiatric Functions: A Mendelian Randomization Analysis
Source: Front Endocrinol (Lausanne). 2019 Aug 16;10:564. doi: 10.3389/fendo.2019.00564 (PMC6706785; doi:10.3389/fendo.2019.00564)
Supplement: Supplementary file 1 [file Data_Sheet_1.docx]

Supplement table 1. Single nucleotide polymorphisms used as instrumental variables for the effect of serum cortisol level on the primary outcome

| SNPs | Effect allele | Effect allele frequency | Serum cortisol ^[1]^  (sd in log scale) | | | Depression symptoms ^[2]^ (score) | |
| --- | --- | --- | --- | --- | --- | --- | --- |
|  |  |  | Beta | se | p | Beta | se |
| rs11621961 | T | 0.36 | -0.08 | 0.01 | 4.41E-08 | 0.007 | 0.004 |
| rs11629171 | T | 0.28 | 0.09 | 0.01 | 6.80E-10 | 0.001 | 0.004 |
| rs7161521 | T | 0.22 | 0.10 | 0.01 | 5.18E-12 | -0.002 | 0.004 |
| rs941599 | T | 0.22 | 0.10 | 0.01 | 4.93E-12 | -0.002 | 0.004 |
| rs2281518 | A | 0.80 | -0.10 | 0.01 | 5.22E-12 | 0.001 | 0.004 |
| rs12589136 | T | 0.22 | 0.10 | 0.01 | 4.04E-12 | -0.002 | 0.004 |
| rs2749530 | A | 0.54 | -0.07 | 0.01 | 4.60E-09 | -0.003 | 0.004 |
| rs2749529 | T | 0.47 | -0.07 | 0.01 | 3.01E-09 | 0.003 | 0.004 |
| rs2749527 | T | 0.49 | 0.08 | 0.01 | 6.21E-11 | 0.000 | 0.003 |
| rs12588394 | T | 0.22 | 0.09 | 0.01 | 1.41E-08 | 0.001 | 0.004 |
| rs3819333 | T | 0.22 | 0.08 | 0.01 | 1.75E-08 | 0.002 | 0.004 |
| rs941595 | T | 0.78 | 0.08 | 0.01 | 3.04E-08 | 0.000 | 0.004 |
| rs1950652 | G | 0.23 | -0.08 | 0.02 | 3.52E-08 | 0.002 | 0.004 |
| rs3762132 | A | 0.54 | -0.07 | 0.01 | 9.57E-09 | -0.006 | 0.004 |
| rs3762130 | C | 0.78 | -0.09 | 0.02 | 1.91E-08 | -0.002 | 0.004 |
| rs3748319 | T | 0.23 | 0.09 | 0.02 | 1.82E-08 | 0.003 | 0.001 |
| rs1243171 | A | 0.53 | -0.07 | 0.01 | 1.08E-08 | 0.005 | 0.004 |

SNPs: Single nucleotide polymorphisms; sd: standard deviation; se: standard error; Beta refers to the association of each variant with the outcome

Supplement table 2. Single nucleotide polymorphisms used as instrumental variables for the effect of salivary cortisol level on the primary outcome

| SNPs | Effect allele | Effect allele frequency | Salivary cortisol ^[3]^  (sd in log scale) | | | Depression symptoms ^[2]^ (score) | |
| --- | --- | --- | --- | --- | --- | --- | --- |
|  |  |  | Beta | se | p | Beta | se |
| rs6768297 | A | 0.94 | -0.34 | 0.06 | 2.01E-07 | -0.013 | 0.008 |
| rs1170109 | T | 0.12 | 0.12 | 0.02 | 3.95E-07 | -0.006 | 0.006 |

SNPs: Single nucleotide polymorphisms; sd: standard deviation; se: standard error; Beta refers to the association of each variant with the outcome

Supplement table 3. Check assumption 2: Pleiotropic associations with potential known confounders

| SNPs | p value of BMI ^[4]^ | p value of Type 2 diabetes ^[5]^ |
| --- | --- | --- |
| rs11621961 | 0.826 | 0.54 |
| rs11629171 | 0.609 | 0.55 |
| rs7161521 | 0.824 | 0.76 |
| rs941599 | 0.859 | 0.76 |
| rs2281518 | 0.859 | 0.73 |
| rs12589136 | 0.807 | 0.68 |
| rs2749530 | 0.739 | 0.88 |
| rs2749529 | 0.697 | 0.92 |
| rs2749527 | 0.803 | 0.73 |
| rs12588394 | 0.689 | 0.74 |
| rs3819333 | 0.673 | 0.88 |
| rs941595 | 0.706 | 0.72 |
| rs1950652 | 0.549 | 0.90 |
| rs3762132 | 0.437 | 0.76 |
| rs3762130 | 0.505 | 0.94 |
| rs3748319 | 0.505 | 0.93 |
| rs1243171 | 0.470 | 0.86 |

SNPs: Single nucleotide polymorphisms; BMI: body mass index

Supplement table 4. Check assumption 3: The association of 261SNPs in the same linkage disequilibrium block with the primary outcome ^[2]^

| SNPs | Chromosome | Position | A1 | Effect allele frequency | p |
| --- | --- | --- | --- | --- | --- |
| rs72706253 | 14 | 94833092 | A | 0.0541 | 0.01108 |
| rs1243165 | 14 | 94844305 | T | 0.1903 | 0.01257 |
| rs41301763 | 14 | 94832898 | T | 0.0541 | 0.01273 |
| rs1243162 | 14 | 94846943 | A | 0.8097 | 0.01399 |
| rs145371730 | 14 | 94833597 | A | 0.0541 | 0.01483 |
| rs72706209 | 14 | 94815157 | A | 0.05037 | 0.01562 |
| rs72704393 | 14 | 94808884 | A | 0.05037 | 0.02032 |
| rs116182924 | 14 | 94828975 | C | 0.94403 | 0.02619 |
| rs145099667 | 14 | 94828122 | A | 0.05597 | 0.02619 |
| rs72706227 | 14 | 94826309 | A | 0.05597 | 0.02655 |
| rs6647 | 14 | 94847415 | A | 0.8097 | 0.02661 |
| rs11846355 | 14 | 94827959 | T | 0.05597 | 0.02706 |
| rs67412635 | 14 | 94826485 | T | 0.94403 | 0.0276 |
| rs142075098 | 14 | 94836611 | A | 0.0597 | 0.02894 |
| rs10144771 | 14 | 94778653 | A | 0.2873 | 0.02895 |
| rs76485178 | 14 | 94836194 | A | 0.0597 | 0.03134 |
| rs1243164 | 14 | 94844470 | A | 0.1903 | 0.03136 |
| rs72706268 | 14 | 94836059 | A | 0.9403 | 0.03141 |
| rs67003893 | 14 | 94837426 | T | 0.9403 | 0.03621 |
| rs67441971 | 14 | 94837211 | C | 0.0597 | 0.03919 |
| rs147569134 | 14 | 94835859 | A | 0.0597 | 0.04703 |
| rs12893029 | 14 | 94795018 | T | 0.2985 | 0.05242 |
| rs72704356 | 14 | 94778663 | T | 0.02799 | 0.056 |
| rs72704358 | 14 | 94779725 | T | 0.03731 | 0.05734 |
| rs149108558 | 14 | 94776771 | T | 0.02799 | 0.05775 |
| rs17783921 | 14 | 94794249 | A | 0.2985 | 0.05776 |
| rs112209637 | 14 | 94778064 | C | 0.97201 | 0.0632 |
| rs12888205 | 14 | 94794672 | C | 0.7015 | 0.06325 |
| rs72704357 | 14 | 94778878 | A | 0.02799 | 0.06404 |
| rs10141488 | 14 | 94777280 | A | 0.02799 | 0.06451 |
| rs72704366 | 14 | 94790627 | T | 0.04104 | 0.06514 |
| rs72704364 | 14 | 94789992 | A | 0.04104 | 0.06537 |
| rs72704349 | 14 | 94775926 | C | 0.97201 | 0.06597 |
| rs1885065 | 14 | 94841817 | T | 0.7463 | 0.06658 |
| rs17783915 | 14 | 94794218 | T | 0.2985 | 0.06674 |
| rs10141408 | 14 | 94777361 | T | 0.02799 | 0.06913 |
| rs72704361 | 14 | 94785459 | T | 0.03545 | 0.07148 |
| rs72704359 | 14 | 94783780 | T | 0.96455 | 0.07385 |
| rs61980636 | 14 | 94784618 | T | 0.1791 | 0.08047 |
| rs72704360 | 14 | 94784123 | T | 0.03545 | 0.08309 |
| rs72704373 | 14 | 94794231 | T | 0.04104 | 0.08331 |
| rs2736887 | 14 | 94812980 | C | 0.1791 | 0.08368 |
| rs1998057 | 14 | 94789192 | A | 0.6119 | 0.08618 |
| rs12880218 | 14 | 94835046 | T | 0.2164 | 0.09353 |
| rs1243167 | 14 | 94841641 | A | 0.2164 | 0.0981 |
| rs1016686 | 14 | 94839240 | T | 0.2164 | 0.1017 |
| rs61280460 | 14 | 94796184 | A | 0.8228 | 0.1101 |
| rs1243166 | 14 | 94843818 | A | 0.3731 | 0.1132 |
| rs67994395 | 14 | 94829527 | T | 0.2761 | 0.1245 |
| rs142074953 | 14 | 94804674 | A | 0.04104 | 0.1252 |
| rs2070709 | 14 | 94846892 | A | 0.2463 | 0.1275 |
| rs17580 | 14 | 94847262 | A | 0.0541 | 0.1308 |
| rs965344 | 14 | 94818078 | A | 0.1791 | 0.1315 |
| rs2073333 | 14 | 94844562 | T | 0.2407 | 0.1332 |
| rs17090719 | 14 | 94846661 | T | 0.7127 | 0.1408 |
| rs1243169 | 14 | 94838740 | A | 0.709 | 0.1483 |
| rs7154770 | 14 | 94782798 | T | 0.7407 | 0.1484 |
| rs61980638 | 14 | 94792848 | A | 0.681 | 0.1545 |
| rs75255245 | 14 | 94806057 | A | 0.05784 | 0.1597 |
| rs2749525 | 14 | 94828584 | C | 0.2164 | 0.1623 |
| rs3827895 | 14 | 94790343 | T | 0.416 | 0.1623 |
| rs72704352 | 14 | 94776994 | A | 0.07836 | 0.1738 |
| rs11846959 | 14 | 94846316 | A | 0.2388 | 0.1788 |
| rs4990242 | 14 | 94828271 | A | 0.2761 | 0.1805 |
| rs11160168 | 14 | 94776401 | T | 0.291 | 0.1893 |
| rs2749534 | 14 | 94809760 | A | 0.7817 | 0.1923 |
| rs12147157 | 14 | 94790801 | A | 0.6866 | 0.1943 |
| rs8005719 | 14 | 94777500 | T | 0.291 | 0.1947 |
| rs941597 | 14 | 94813361 | A | 0.2183 | 0.2033 |
| rs1956175 | 14 | 94810258 | A | 0.2183 | 0.2116 |
| rs2281520 | 14 | 94776387 | A | 0.7071 | 0.2164 |
| rs1243173 | 14 | 94836298 | A | 0.4515 | 0.2176 |
| rs2736888 | 14 | 94810725 | C | 0.7817 | 0.22 |
| rs2736897 | 14 | 94824168 | T | 0.7836 | 0.2212 |
| rs4905192 | 14 | 94829062 | T | 0.2183 | 0.2246 |
| rs72706230 | 14 | 94827653 | A | 0.05224 | 0.2247 |
| rs2736895 | 14 | 94824852 | A | 0.7836 | 0.2262 |
| rs1815228 | 14 | 94811163 | T | 0.2183 | 0.2291 |
| rs2736896 | 14 | 94824340 | A | 0.2164 | 0.2293 |
| rs1303 | 14 | 94844843 | T | 0.7575 | 0.232 |
| rs11160172 | 14 | 94829998 | T | 0.2146 | 0.2331 |
| rs2736885 | 14 | 94815476 | A | 0.7799 | 0.2351 |
| rs2250095 | 14 | 94813351 | C | 0.2183 | 0.2355 |
| rs2749533 | 14 | 94810097 | A | 0.2183 | 0.2368 |
| rs2749532 | 14 | 94814819 | A | 0.7817 | 0.241 |
| rs4905193 | 14 | 94829303 | T | 0.2164 | 0.2412 |
| rs2144833 | 14 | 94773788 | T | 0.2929 | 0.2418 |
| rs2736901 | 14 | 94820806 | T | 0.2239 | 0.242 |
| rs2749526 | 14 | 94828491 | A | 0.2313 | 0.2425 |
| rs17090693 | 14 | 94841331 | T | 0.7612 | 0.2477 |
| rs2749528 | 14 | 94820864 | T | 0.2705 | 0.2483 |
| rs79249070 | 14 | 94835960 | A | 0.4496 | 0.2513 |
| rs1956178 | 14 | 94786544 | A | 0.1623 | 0.2555 |
| rs2228542 | 14 | 94776219 | T | 0.2929 | 0.2628 |
| rs2003640 | 14 | 94814284 | T | 0.2985 | 0.2644 |
| rs1956173 | 14 | 94816240 | A | 0.09701 | 0.2717 |
| rs61980637 | 14 | 94792445 | A | 0.8377 | 0.2751 |
| rs715033 | 14 | 94790638 | T | 0.8377 | 0.2767 |
| rs1243168 | 14 | 94841366 | A | 0.2463 | 0.2813 |
| rs1042394 | 14 | 94772504 | A | 0.2929 | 0.2909 |
| rs1956172 | 14 | 94833033 | T | 0.2146 | 0.2937 |
| rs1815227 | 14 | 94831814 | A | 0.2146 | 0.2952 |
| rs17090691 | 14 | 94840201 | A | 0.8694 | 0.2969 |
| rs926144 | 14 | 94813402 | A | 0.8209 | 0.3043 |
| rs59173829 | 14 | 94774023 | A | 0.7052 | 0.3083 |
| rs61980587 | 14 | 94774186 | T | 0.7052 | 0.3102 |
| rs1998056 | 14 | 94789495 | C | 0.416 | 0.3191 |
| rs7161231 | 14 | 94808760 | T | 0.1175 | 0.3426 |
| rs6575422 | 14 | 94833807 | T | 0.2146 | 0.344 |
| rs1950654 | 14 | 94805411 | T | 0.7463 | 0.3462 |
| rs9944117 | 14 | 94841989 | T | 0.1306 | 0.3592 |
| rs1051052 | 14 | 94843932 | A | 0.3769 | 0.3651 |
| rs877084 | 14 | 94842769 | A | 0.1325 | 0.3664 |
| rs875989 | 14 | 94842145 | T | 0.1306 | 0.3699 |
| rs877083 | 14 | 94842842 | A | 0.1306 | 0.3713 |
| rs1884546 | 14 | 94841824 | T | 0.1306 | 0.3726 |
| rs877082 | 14 | 94842926 | A | 0.1306 | 0.3746 |
| rs35087450 | 14 | 94778307 | T | 0.7332 | 0.3791 |
| rs4905179 | 14 | 94795492 | A | 0.8228 | 0.394 |
| rs11626522 | 14 | 94781461 | A | 0.07276 | 0.3966 |
| rs12437224 | 14 | 94773395 | T | 0.1791 | 0.3978 |
| rs56349806 | 14 | 94800222 | T | 0.07463 | 0.3979 |
| rs2144835 | 14 | 94773411 | T | 0.7239 | 0.3996 |
| rs4900225 | 14 | 94788593 | A | 0.4328 | 0.4 |
| rs1884549 | 14 | 94841500 | A | 0.8694 | 0.402 |
| rs3748320 | 14 | 94780608 | A | 0.2593 | 0.4193 |
| rs1956177 | 14 | 94789810 | T | 0.2537 | 0.4213 |
| rs4900230 | 14 | 94839102 | A | 0.6754 | 0.4258 |
| rs34097828 | 14 | 94787674 | C | 0.7276 | 0.4285 |
| rs34025389 | 14 | 94809810 | T | 0.03172 | 0.4491 |
| rs1884548 | 14 | 94841670 | A | 0.1325 | 0.4529 |
| rs877081 | 14 | 94843083 | A | 0.1306 | 0.4541 |
| rs996050 | 14 | 94801368 | T | 0.4757 | 0.4626 |
| rs4592582 | 14 | 94832796 | A | 0.07276 | 0.4674 |
| rs11832 | 14 | 94843565 | T | 0.4907 | 0.4708 |
| rs2736899 | 14 | 94823220 | T | 0.5522 | 0.4724 |
| rs75199535 | 14 | 94807794 | A | 0.05784 | 0.4738 |
| rs4905194 | 14 | 94836471 | C | 0.2257 | 0.4852 |
| rs1884547 | 14 | 94841818 | A | 0.5336 | 0.4877 |
| rs7158343 | 14 | 94783153 | C | 0.7257 | 0.4905 |
| rs8021983 | 14 | 94792549 | A | 0.7332 | 0.4931 |
| rs2013150 | 14 | 94825769 | A | 0.4459 | 0.4961 |
| rs78533181 | 14 | 94825350 | A | 0.0597 | 0.4964 |
| rs11627241 | 14 | 94785451 | T | 0.2519 | 0.5014 |
| rs6575414 | 14 | 94791534 | A | 0.7463 | 0.5048 |
| rs59036614 | 14 | 94830448 | A | 0.4459 | 0.5169 |
| rs56044130 | 14 | 94821812 | A | 0.92537 | 0.5177 |
| rs941600 | 14 | 94788310 | T | 0.2537 | 0.5196 |
| rs56306384 | 14 | 94814013 | T | 0.07463 | 0.5207 |
| rs4905177 | 14 | 94788715 | T | 0.2537 | 0.5236 |
| rs56045385 | 14 | 94829923 | A | 0.2201 | 0.529 |
| rs2092977 | 14 | 94783172 | A | 0.2519 | 0.5294 |
| rs12590834 | 14 | 94783793 | A | 0.1381 | 0.5376 |
| rs78739778 | 14 | 94787072 | C | 0.7463 | 0.5409 |
| rs3762127 | 14 | 94835462 | A | 0.2313 | 0.5504 |
| rs17751614 | 14 | 94841542 | T | 0.1082 | 0.5547 |
| rs760335 | 14 | 94814944 | T | 0.4795 | 0.5597 |
| rs3790036 | 14 | 94773121 | A | 0.834 | 0.5637 |
| rs72704401 | 14 | 94811520 | A | 0.07463 | 0.5683 |
| rs11628917 | 14 | 94843719 | T | 0.1082 | 0.57 |
| rs72704338 | 14 | 94772925 | T | 0.8209 | 0.5711 |
| rs71431637 | 14 | 94839421 | A | 0.93284 | 0.5715 |
| rs79867353 | 14 | 94811564 | A | 0.07463 | 0.5721 |
| rs2736883 | 14 | 94815785 | T | 0.4291 | 0.5759 |
| rs72704399 | 14 | 94811210 | C | 0.92164 | 0.58 |
| rs71431631 | 14 | 94775707 | T | 0.2649 | 0.5816 |
| rs79247904 | 14 | 94776034 | A | 0.01679 | 0.5833 |
| rs56252611 | 14 | 94789223 | A | 0.07463 | 0.5865 |
| rs11627377 | 14 | 94815887 | T | 0.6996 | 0.5871 |
| rs8005533 | 14 | 94777406 | T | 0.584 | 0.589 |
| rs10498639 | 14 | 94775526 | A | 0.4123 | 0.5952 |
| rs34362280 | 14 | 94831028 | C | 0.4235 | 0.5963 |
| rs7161291 | 14 | 94831547 | A | 0.08582 | 0.5998 |
| rs11160169 | 14 | 94776441 | A | 0.416 | 0.6033 |
| rs72704334 | 14 | 94772851 | T | 0.8209 | 0.6138 |
| rs12437272 | 14 | 94773371 | T | 0.1791 | 0.623 |
| rs60271218 | 14 | 94818931 | A | 0.7705 | 0.6426 |
| rs58622098 | 14 | 94825670 | A | 0.2276 | 0.6436 |
| rs1950657 | 14 | 94804737 | A | 0.5224 | 0.6456 |
| rs2736889 | 14 | 94809300 | A | 0.569 | 0.6555 |
| rs2228543 | 14 | 94772429 | A | 0.1791 | 0.658 |
| rs2736890 | 14 | 94827409 | T | 0.4235 | 0.6619 |
| rs61981771 | 14 | 94826068 | A | 0.98134 | 0.6627 |
| rs2749540 | 14 | 94803186 | A | 0.4757 | 0.6666 |
| rs2736892 | 14 | 94802731 | A | 0.3284 | 0.6706 |
| rs76221089 | 14 | 94841855 | A | 0.08769 | 0.6737 |
| rs2281519 | 14 | 94776632 | A | 0.2537 | 0.6806 |
| rs58776570 | 14 | 94824138 | A | 0.7724 | 0.683 |
| rs11568814 | 14 | 94843455 | T | 0.08955 | 0.6835 |
| rs11160173 | 14 | 94833689 | T | 0.2799 | 0.6866 |
| rs55980335 | 14 | 94824484 | T | 0.2276 | 0.6885 |
| rs1950658 | 14 | 94804293 | A | 0.3284 | 0.6903 |
| rs2749541 | 14 | 94802453 | C | 0.6716 | 0.6926 |
| rs7145181 | 14 | 94823828 | A | 0.2276 | 0.6949 |
| rs1810389 | 14 | 94814462 | A | 0.5205 | 0.7003 |
| rs2749544 | 14 | 94799522 | A | 0.4757 | 0.7007 |
| rs56872560 | 14 | 94817669 | A | 0.2295 | 0.7023 |
| rs7148985 | 14 | 94815268 | T | 0.1175 | 0.7038 |
| rs1950656 | 14 | 94805094 | A | 0.3284 | 0.7049 |
| rs35854995 | 14 | 94827985 | T | 0.2108 | 0.7051 |
| rs76424594 | 14 | 94833216 | T | 0.07276 | 0.7055 |
| rs4905191 | 14 | 94828841 | A | 0.7705 | 0.706 |
| rs941596 | 14 | 94814999 | T | 0.4795 | 0.7063 |
| rs4283161 | 14 | 94828053 | T | 0.8396 | 0.7067 |
| rs2749542 | 14 | 94800241 | T | 0.5243 | 0.7083 |
| rs2749543 | 14 | 94800054 | A | 0.4757 | 0.7143 |
| rs1243163 | 14 | 94844706 | A | 0.03731 | 0.7214 |
| rs113375097 | 14 | 94828074 | T | 0.181 | 0.7292 |
| rs4900227 | 14 | 94831363 | C | 0.5392 | 0.7307 |
| rs2005945 | 14 | 94814300 | T | 0.5821 | 0.7336 |
| rs1810388 | 14 | 94814466 | A | 0.4757 | 0.7369 |
| rs996051 | 14 | 94801350 | T | 0.6716 | 0.7388 |
| rs746531 | 14 | 94797308 | A | 0.3284 | 0.7421 |
| rs2736884 | 14 | 94815481 | T | 0.5205 | 0.745 |
| rs746530 | 14 | 94797293 | A | 0.3284 | 0.7452 |
| rs2736886 | 14 | 94815400 | A | 0.4795 | 0.7496 |
| rs1956179 | 14 | 94785742 | T | 0.7276 | 0.774 |
| rs941598 | 14 | 94811470 | T | 0.4291 | 0.779 |
| rs1806364 | 14 | 94801876 | A | 0.3284 | 0.7852 |
| rs4905187 | 14 | 94805193 | T | 0.1959 | 0.7888 |
| rs6575415 | 14 | 94791601 | A | 0.1959 | 0.7926 |
| rs10129500 | 14 | 94796319 | A | 0.3284 | 0.8033 |
| rs4491436 | 14 | 94804700 | A | 0.8041 | 0.8053 |
| rs11629326 | 14 | 94808831 | A | 0.7724 | 0.8055 |
| rs2281517 | 14 | 94789787 | A | 0.8022 | 0.8099 |
| rs1961119 | 14 | 94835959 | T | 0.4795 | 0.819 |
| rs76961279 | 14 | 94783374 | T | 0.93284 | 0.8373 |
| rs1956174 | 14 | 94816121 | A | 0.2257 | 0.8421 |
| rs718187 | 14 | 94801860 | T | 0.8041 | 0.8467 |
| rs116166665 | 14 | 94829217 | T | 0.1157 | 0.8558 |
| rs9989237 | 14 | 94795202 | T | 0.1959 | 0.8591 |
| rs941594 | 14 | 94835914 | A | 0.5093 | 0.8656 |
| rs2749527 | 14 | 94827068 | T | 0.4981 | 0.8845 |
| rs8015996 | 14 | 94772157 | A | 0.2519 | 0.8864 |
| rs2736894 | 14 | 94799376 | A | 0.3265 | 0.8874 |
| rs3790035 | 14 | 94773242 | T | 0.2519 | 0.8911 |
| rs11623050 | 14 | 94833567 | C | 0.278 | 0.8971 |
| rs8023023 | 14 | 94773876 | A | 0.4123 | 0.9043 |
| rs11622970 | 14 | 94775737 | A | 0.5858 | 0.907 |
| rs80140444 | 14 | 94785380 | A | 0.98321 | 0.9127 |
| rs17824597 | 14 | 94840376 | C | 0.91791 | 0.9142 |
| rs2180401 | 14 | 94774017 | T | 0.4123 | 0.9142 |
| rs8022616 | 14 | 94773945 | A | 0.91231 | 0.9156 |
| rs2144832 | 14 | 94774192 | T | 0.5877 | 0.9187 |
| rs1950660 | 14 | 94774300 | A | 0.4123 | 0.9191 |
| rs56001064 | 14 | 94840163 | A | 0.08209 | 0.9217 |
| rs11623055 | 14 | 94775701 | A | 0.4123 | 0.928 |
| rs11627651 | 14 | 94775687 | T | 0.5858 | 0.9301 |
| rs2736898 | 14 | 94823817 | T | 0.4963 | 0.9318 |
| rs2228541 | 14 | 94776221 | A | 0.584 | 0.9418 |
| rs4900228 | 14 | 94833371 | A | 0.7313 | 0.9432 |
| rs2144834 | 14 | 94773634 | A | 0.2593 | 0.9458 |
| rs7493065 | 14 | 94818940 | T | 0.2295 | 0.9471 |
| rs6575423 | 14 | 94833997 | A | 0.7295 | 0.955 |
| rs909287 | 14 | 94808664 | A | 0.2276 | 0.9704 |
| rs8010960 | 14 | 94797895 | A | 0.05037 | 0.9831 |
| rs1950661 | 14 | 94774298 | A | 0.5877 | 0.985 |
| rs11622665 | 14 | 94774995 | A | 0.8396 | 0.9858 |
| rs4905188 | 14 | 94821237 | T | 0.7724 | 0.9901 |
| rs41310932 | 14 | 94832739 | A | 0.9459 | 0.9954 |
| rs2749539 | 14 | 94803365 | T | 0.5653 | 0.9969 |

SNPs: Single nucleotide polymorphisms;

Supplement Figure 1. Sensitivity analysis of serum cortisol on primary outcome


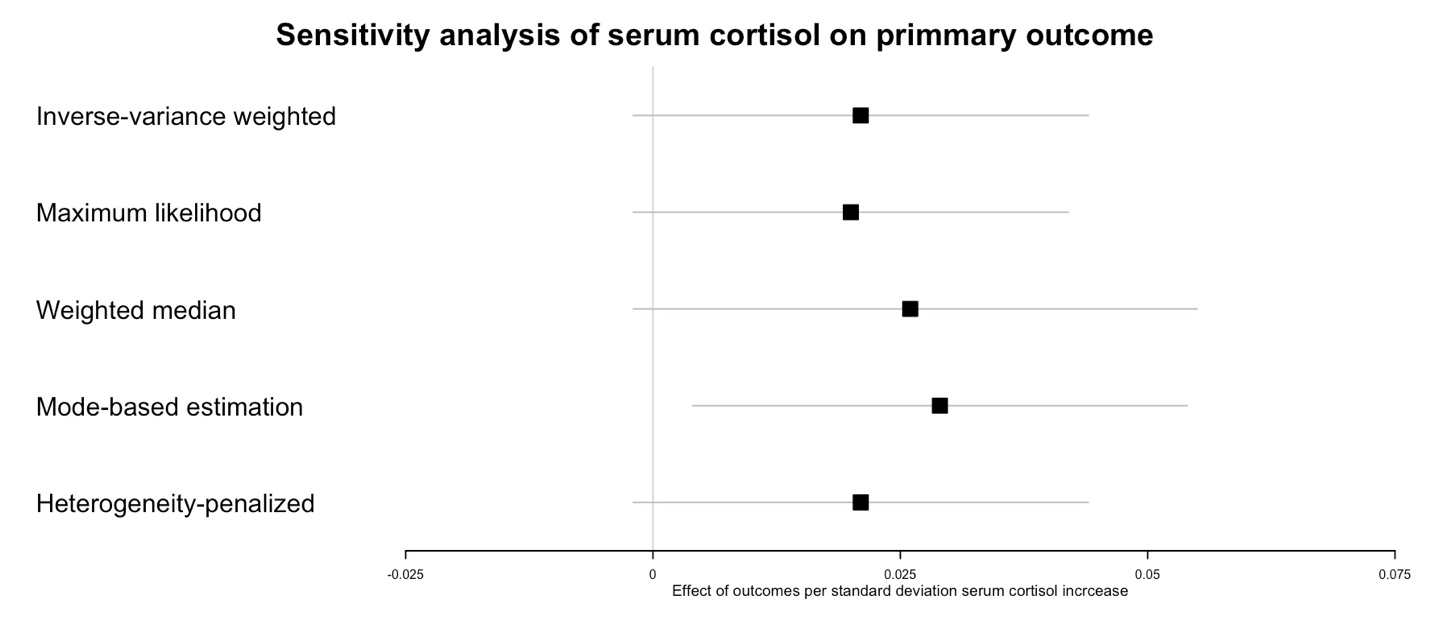


References:

1. Velders FP, Kuningas M, Kumari M, et al. Genetics of cortisol secretion and depressive symptoms: A candidate gene and genome wide association approach. *Psychoneuroendocrinology*. 2011;36(7):1053-1061. doi:10.1016/j.psyneuen.2011.01.003.
2. Okbay A, Baselmans BML, De Neve J-E, et al. Genetic variants associated with subjective well-being, depressive symptoms, and neuroticism identified through genome-wide analyses. *Nat Genet*. 2016;48(6):624-633. doi:10.1038/ng.3552.
3. Neumann A, Direk N, Crawford AA, et al. The low single nucleotide polymorphism heritability of plasma and saliva cortisol levels. *Psychoneuroendocrinology*. 2017;85:88-95. doi:10.1016/j.psyneuen.2017.08.011.
4. Shungin D, Winkler TW, Croteau-Chonka DC, et al. New genetic loci link adipose and insulin biology to body fat distribution. *Nature*. 2015;518(7538):187-196. doi:10.1038/nature14132.
5. Scott RA, Scott LJ, Mägi R, et al. An Expanded Genome-Wide Association Study of Type 2 Diabetes in Europeans. *Diabetes*. 2017;66(11):2888-2902. doi:10.2337/db16-1253.
